# A Christmas Appeal

**Published:** 1920-12-25

**Authors:** 


### A Christmas Appeal.

Lord Wolverton, the President of the Putney Home for Incurables, writes: One is heartbroken to think that there can be in this country such a mass of human suffering, such a mass of irremediable distress. On the other hand, when you think of the angelic ministrations, of the helpful medical and surgical treatment, of the careful nursing, of the general manifestations of sympathy which you see at the Royal Hospital and Home for Incurables, Putney, one really rejoices that, after all, this is not a decadent age, but that we are animated by Christian sympathy and by a lofty conception of individual and public obligations. At the present time this national charity has a heavy overdraft at its bankers, and during the last eighteen months £20,000 from its slender investments have been withdrawn and used. Will the benevolent public please come to our aid this Christmastide? Any gifts sent to the Secretary of the institution at the City offices, Bond Court House, Walbrook, E.C. 4, will be gratefully and promptly acknowledged.

